# Corticosteroid dose reporting in atraumatic osteonecrosis of the humeral head: a scoping review

**DOI:** 10.1016/j.xrrt.2026.100877

**Published:** 2026-09-04

**Authors:** Thomas Ciavarro, Amr Elmaraghy, Patrick J. Carroll

**Affiliations:** a University College Dublin, School of Medicine, Dublin, Ireland; b Division of Orthopaedic Surgery, Department of Surgery, University of Toronto, Toronto, ON, Canada; c St. Joseph's Health Centre, Unity Health Toronto, Toronto, ON, Canada; d Li Ka Shing Knowledge Institute, Toronto, ON, Canada

**Keywords:** Corticosteroids, Steroid-induced osteonecrosis, Avascular necrosis, Humeral head, Shoulder osteonecrosis, Glucocorticoids, Corticosteroid dosage, Scoping review

## Abstract

**Background:**

Steroid-induced avascular necrosis of the humeral head is an uncommon but clinically important complication of corticosteroid exposure. Although steroid-associated osteonecrosis has been extensively studied in the femoral head, considerably less attention has been directed toward the shoulder, particularly regarding whether a dose-response relationship or clinically meaningful corticosteroid threshold exists. This scoping review aimed to map the available literature on steroid-induced humeral head avascular necrosis and evaluate how corticosteroid dose, duration, route of administration, and timing have been reported.

**Methods:**

A structured literature search was conducted across PubMed/MEDLINE, Embase, the Cochrane Library, and Google Scholar in accordance with scoping review methodology. Eligible studies included adult patients with humeral head avascular necrosis associated with corticosteroid exposure. Data were charted regarding study design, patient population, corticosteroid exposure, diagnostic approach, disease stage, management, and reported outcomes.

**Results:**

Fifty-two sources of evidence were included; the literature was limited and heterogeneous, consisting largely of case reports, case series, and retrospective studies. Corticosteroid exposure was frequently identified as a risk factor, but dose reporting was inconsistent. Few studies provided sufficient detail regarding cumulative dose, peak dose, daily dose, route, duration, or timing of therapy. No consistent corticosteroid dose threshold for humeral head avascular necrosis could be identified.

**Conclusion:**

Current evidence suggests an association between corticosteroid exposure and humeral head avascular necrosis, but the literature remains insufficient to define a reliable dose-response relationship or clinically useful dose threshold. Future studies should standardize reporting of corticosteroid exposure, including cumulative dose, peak dose, route, duration, and timing relative to symptom onset or diagnosis.

Osteonecrosis or avascular necrosis of the humeral head is a rare but clinically important disorder caused by compromised osseous blood supply, resulting in osteocyte death, subchondral weakening, and, in advanced disease, articular collapse.^1^ The humeral head is widely recognized as the second most common site of non-traumatic osteonecrosis after the femoral head, yet shoulder-specific disease remains comparatively under-studied.^8^^,^^11^^,^^12^ Corticosteroid exposure is among the most frequently reported non-traumatic risk factors for humeral head osteonecrosis, alongside conditions such as sickle cell disease, alcohol use, dysbarism, and systemic inflammatory disease.^8^^,^^11^^,^^18^ Although corticosteroid-associated osteonecrosis is biologically plausible and well documented, the dose-risk relationship remains uncertain; broader steroid-induced osteonecrosis literature has not reached consensus on whether risk is best predicted by cumulative dose, peak dose, daily dose, route of administration, or duration of therapy.^5^^,^^15^ This uncertainty is especially relevant to the shoulder, where much of the available literature focuses on natural history, imaging, staging, and surgical management rather than detailed corticosteroid exposure.^4^^,^^6^^,^^10^^,^^14^ In the largest shoulder-specific corticosteroid cohort, Hernigou et al^7^ reported that humeral head osteonecrosis was diagnosed a mean of 15 months after corticosteroid initiation and that patients with shoulder involvement had higher cumulative and peak methylprednisolone-equivalent doses than corticosteroid-exposed patients without shoulder involvement. However, a reproducible dose threshold was not established. Therefore, clarification of how corticosteroid dose is reported in the humeral head osteonecrosis literature is necessary before any clinically meaningful threshold can be proposed.

The available literature on corticosteroid-associated humeral head osteonecrosis remains fragmented, consisting mainly of case reports, retrospective series, surgical outcome studies, and narrative or systematic reviews.^6^^,^^11^^,^^12^ Although many shoulder-focused studies identify steroid therapy as a contributing etiologic factor, few describe exposure in enough detail to permit comparison across reports.^15^^,^^18^ Studies also differ in patient population, indication for steroid treatment, route of administration, imaging modality, disease stage, duration of follow-up, and whether shoulder involvement occurred in isolation or as part of multifocal osteonecrosis.^2^^,^^8^^,^^11^ Because of this clinical and methodological variability, the evidence is poorly suited to meta-analysis. A scoping review was therefore used to map how steroid dose has been documented, determine whether dose-response patterns have been examined, and clarify whether the literature supports any reproducible exposure threshold for atraumatic osteonecrosis of the humeral head.

The aim of this scoping review was to map the available adult clinical literature on corticosteroid exposure and atraumatic osteonecrosis of the humeral head. Specifically, this review examined how corticosteroid dose has been reported, whether dose-response relationships have been evaluated, and whether any reproducible dose threshold has been proposed for the development of humeral head osteonecrosis.

## Materials and methods

### Study design

This review used a scoping methodology to characterize the extent, nature, and reporting patterns of the adult clinical literature on steroid exposure and atraumatic osteonecrosis of the humeral head. The study was exempt from institutional review board approval because it was a scoping review of previously published literature and did not involve human participants, patient contact, identifiable private data, or animal subjects. This approach was selected because the evidence base varied substantially in study design, patient population, corticosteroid indication, imaging criteria, exposure reporting, and clinical outcomes. Reporting was guided by the Preferred Reporting Items for Systematic Reviews and Meta-Analyses extension for Scoping Reviews checklist.^20^

### Research question

The primary research question was as follows: What evidence exists regarding corticosteroid dose and the development of atraumatic osteonecrosis of the humeral head in adults? Secondary questions examined which corticosteroid dose characteristics were reported, including cumulative dose, peak dose, daily dose, treatment duration, route of administration, and use of pulse therapy. The review also assessed whether included studies evaluated a dose-response relationship or proposed a corticosteroid dose threshold for the development of humeral head osteonecrosis.

### Eligibility criteria

Studies were eligible for inclusion if they reported adult patients aged 18 years or older with atraumatic osteonecrosis of the humeral head and documented corticosteroid exposure. Eligible study designs included cohort studies, case-control studies, case series, case reports, systematic reviews, and narrative reviews where shoulder-specific clinical data were available. Studies were excluded if they involved traumatic humeral head osteonecrosis, pediatric-only populations, animal or basic science studies without clinical data, or osteonecrosis limited to anatomical sites other than the humeral head. Studies reporting multifocal osteonecrosis were eligible only when humeral head involvement was explicitly reported.

### Information sources and search strategy

A structured literature search was conducted using PubMed/MEDLINE, Embase, the Cochrane Library, and Google Scholar to identify studies addressing corticosteroid exposure and atraumatic osteonecrosis of the humeral head. PubMed/MEDLINE and Embase searches were finalized on May 18, 2026. The Cochrane Library and Google Scholar searches were updated on July 16, 2026. These sources were selected to capture indexed biomedical literature, systematic review and trial literature, and additional academic records that may not be readily identified through a single database. Search strategies were developed around the following 3 core concepts: osteonecrosis, humeral head or shoulder involvement, and corticosteroid exposure. A broader shoulder-specific search and a dose-specific search were performed in PubMed/MEDLINE and Embase to capture both general humeral head osteonecrosis literature and studies specifically addressing dose, cumulative dose, dose-response, or threshold reporting.

The full search strings used for each database were as follows:

PubMed/MEDLINE broad search:

(“osteonecrosis” OR “avascular necrosis” OR “aseptic necrosis”) AND (“humeral head” OR “shoulder” OR “proximal humerus”) AND (“corticosteroid” OR “glucocorticoid” OR “steroid” OR “steroid-induced”).

PubMed/MEDLINE dose-specific search:

(“osteonecrosis” OR “avascular necrosis” OR “aseptic necrosis”) AND (“humeral head” OR “shoulder” OR “proximal humerus”) AND (“corticosteroid” OR “glucocorticoid” OR “steroid” OR “steroid-induced”) AND (“dose” OR “cumulative dose” OR “dose-response” OR “threshold”).

Embase broad search:

(‘osteonecrosis’ OR ‘avascular necrosis’ OR ‘aseptic necrosis’) AND (‘humeral head’ OR ‘shoulder’ OR ‘proximal humerus’) AND (‘corticosteroid’ OR ‘glucocorticoid’ OR ‘steroid’ OR ‘steroid-induced’).

Embase dose-specific search:

(‘osteonecrosis’:ti,ab,kw OR ‘avascular necrosis’:ti,ab,kw OR ‘aseptic necrosis’:ti,ab,kw) AND (‘humeral head’:ti,ab,kw OR ‘shoulder’:ti,ab,kw OR ‘proximal humerus’:ti,ab,kw) AND (‘corticosteroid’:ti,ab,kw OR ‘glucocorticoid’:ti,ab,kw OR ‘steroid':ti,ab,kw OR ‘steroid-induced’:ti,ab,kw) AND (‘dose’:ti,ab,kw OR ‘cumulative dose’:ti,ab,kw OR ‘dose-response’:ti,ab,kw OR ‘threshold’:ti,ab,kw).

Cochrane Library:

#1 (osteonecrosis OR “avascular necrosis” OR “aseptic necrosis”):ti,ab,kw.

#2 (“humeral head” OR shoulder OR “proximal humerus”):ti,ab,kw.

#3 (corticosteroid OR glucocorticoid OR steroid OR “steroid-induced”):ti,ab,kw.

#4 #1 AND #2 AND #3.

Google Scholar:

(“osteonecrosis” OR “avascular necrosis” OR “aseptic necrosis”) (“humeral head” OR shoulder OR “proximal humerus”) (corticosteroid OR glucocorticoid OR steroid OR “steroid-induced”).

The PubMed/MEDLINE broad search identified 90 records, and the PubMed/MEDLINE dose-specific search identified 21 records. The Embase broad search identified 425 records, and the Embase dose-specific search identified 26 records. The updated Cochrane Library search identified 5 records, of which 1 was eligible for inclusion. The updated Google Scholar search retrieved 35 records. After removal of 2 duplicate records within the export and 4 records already identified through other sources, 29 new records were screened; 22 were excluded and 7 were included. Reference lists of included articles and relevant review papers were also manually screened to identify additional eligible studies.

### Study selection

The database searches identified 567 records as follows: 111 from PubMed/MEDLINE, 451 from Embase, and 5 from the Cochrane Library. After removal of 125 duplicate records, 442 unique records remained for screening. Following title and abstract screening, 391 records were excluded, leaving 51 reports for eligibility assessment. All 51 reports were retrieved, and 6 were excluded after eligibility review, resulting in 45 database-derived sources being included. Google Scholar was evaluated separately as a supplementary source. The updated search retrieved 35 records; after removal of 2 duplicates within the export and 4 overlaps with previously identified records, 29 new records were screened. Twenty-two were excluded and 7 were included. The final evidence set therefore consisted of 52 unique sources. The study identification, screening, and inclusion process is summarized in the Preferred Reporting Items for Systematic Reviews and Meta-Analyses 2020 flow diagram (Fig. 1).

**Figure 1:**
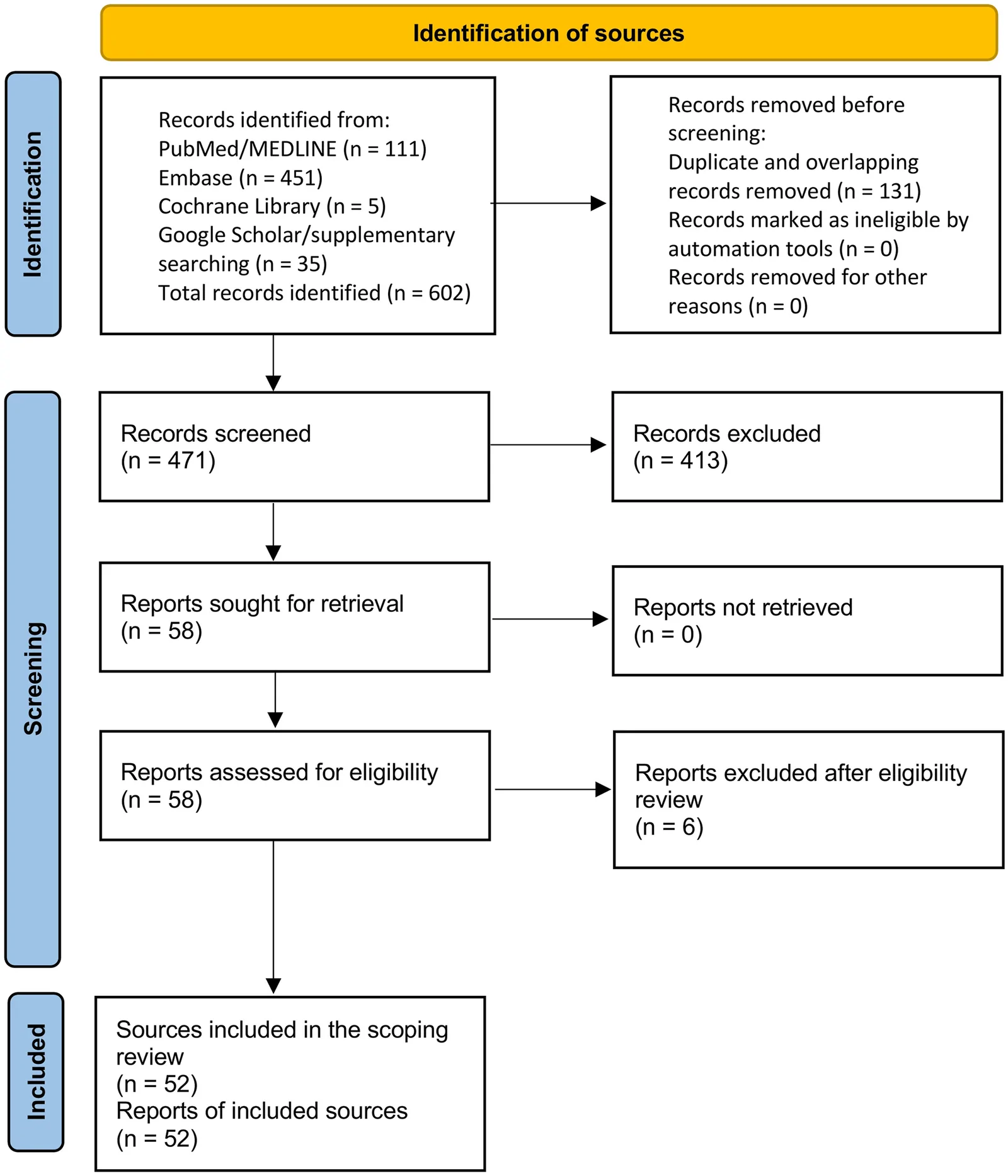
PRISMA 2020 flow diagram of source identification, screening, and inclusion. *PRISMA*, Preferred Reporting Items for Systematic Reviews and Meta-Analyses.

### Data charting

Data from included sources were charted using a standardized extraction table developed for this review. Extracted variables included author, year of publication, journal, country, study design, level of evidence, number of patients, number of shoulders, steroid indication, steroid type, route of administration, cumulative dose, peak dose, daily dose, duration of therapy, use of pulse therapy, timing from steroid exposure to osteonecrosis diagnosis, site of osteonecrosis, disease stage at diagnosis, presence of humeral head collapse, surgical treatment, whether dose-response was analyzed, whether a corticosteroid threshold was proposed, reported confounders, and key study conclusions. Particular attention was given to whether corticosteroid exposure was reported quantitatively and whether the reported dose data were usable for dose-response interpretation. Extracted data were reviewed for consistency, and uncertainties regarding study categorization or dose interpretation were resolved through full-text reassessment and discussion with the supervising investigator.

### Synthesis of results

Findings were synthesized narratively because the included studies were expected to vary substantially in design, population, corticosteroid indication, dose reporting, imaging criteria, follow-up duration, and clinical outcomes.

Studies were grouped into the following two evidence categories: shoulder-specific clinical or review studies reporting humeral head osteonecrosis and case reports or multifocal osteonecrosis reports in which humeral head involvement and corticosteroid exposure were explicitly reported. The synthesis focused on patterns of dose reporting, including whether studies reported cumulative dose, peak dose, daily dose, duration, route of administration, pulse therapy, dose-response analysis, or a proposed dose threshold. No meta-analysis was performed because of heterogeneity in study design and inconsistent reporting of corticosteroid exposure.

Included sources were charted by evidence category, anatomical relevance, study design, corticosteroid exposure reporting, dose quantification, route, duration, and whether a threshold was proposed. Sources identified through Google Scholar or supplementary/manual searching were reconciled against the Rayyan-included database records to avoid duplicate counting. Descriptive counts were generated for evidence category and corticosteroid dose-reporting completeness across the final 52-source evidence set. No formal critical appraisal or risk-of-bias assessment was performed because the purpose of this scoping review was to map the available evidence and characterize reporting patterns rather than to estimate a pooled treatment effect.

### Dose-reporting classification

Corticosteroid dose reporting was categorized at the source level. “Quantified corticosteroid dose reported” was assigned when a source provided extractable numerical steroid exposure data, such as cumulative dose, peak dose, daily dose, injection dose, or equivalent-dose conversion. “Partial/incomplete corticosteroid dose data” was assigned when steroid exposure was described with some numerical detail but was insufficient for full interpretation, such as reporting dose without duration or route, or describing treatment intensity without a cumulative or daily value. “Steroid exposure mentioned but not quantified” was assigned when corticosteroid use was identified as an etiologic or associated factor, but no extractable numerical dose data were provided. “No extractable corticosteroid dose data” was assigned when steroid exposure could not be interpreted from the source for the purpose of dose-response assessment. Sources were also charted for whether a dose-response relationship was evaluated and whether a dose threshold was proposed.

## Results

### Characteristics of included evidence

The final evidence set included 52 unique sources (Fig. 2). The shoulder-specific literature included a randomized study, retrospective case series, radiographic progression studies, rehabilitation studies, core decompression series, arthroplasty outcome studies, systematic reviews, meta-analyses, narrative reviews, and current-concepts reviews. Most studies focused on natural history, staging, imaging, rehabilitation, core decompression, arthroplasty outcomes, or clinical management rather than quantitative corticosteroid exposure.^4^^,^^6^^,^^8^^,^^10^, ^11^, ^12^^,^^14^^,^^18^ Case reports and multifocal osteonecrosis reports more frequently provided individual-level steroid exposure details, although their designs did not permit estimation of incidence, relative risk, or a reliable dose threshold.^1^, ^2^, ^3^^,^^19^

**Figure 2:**
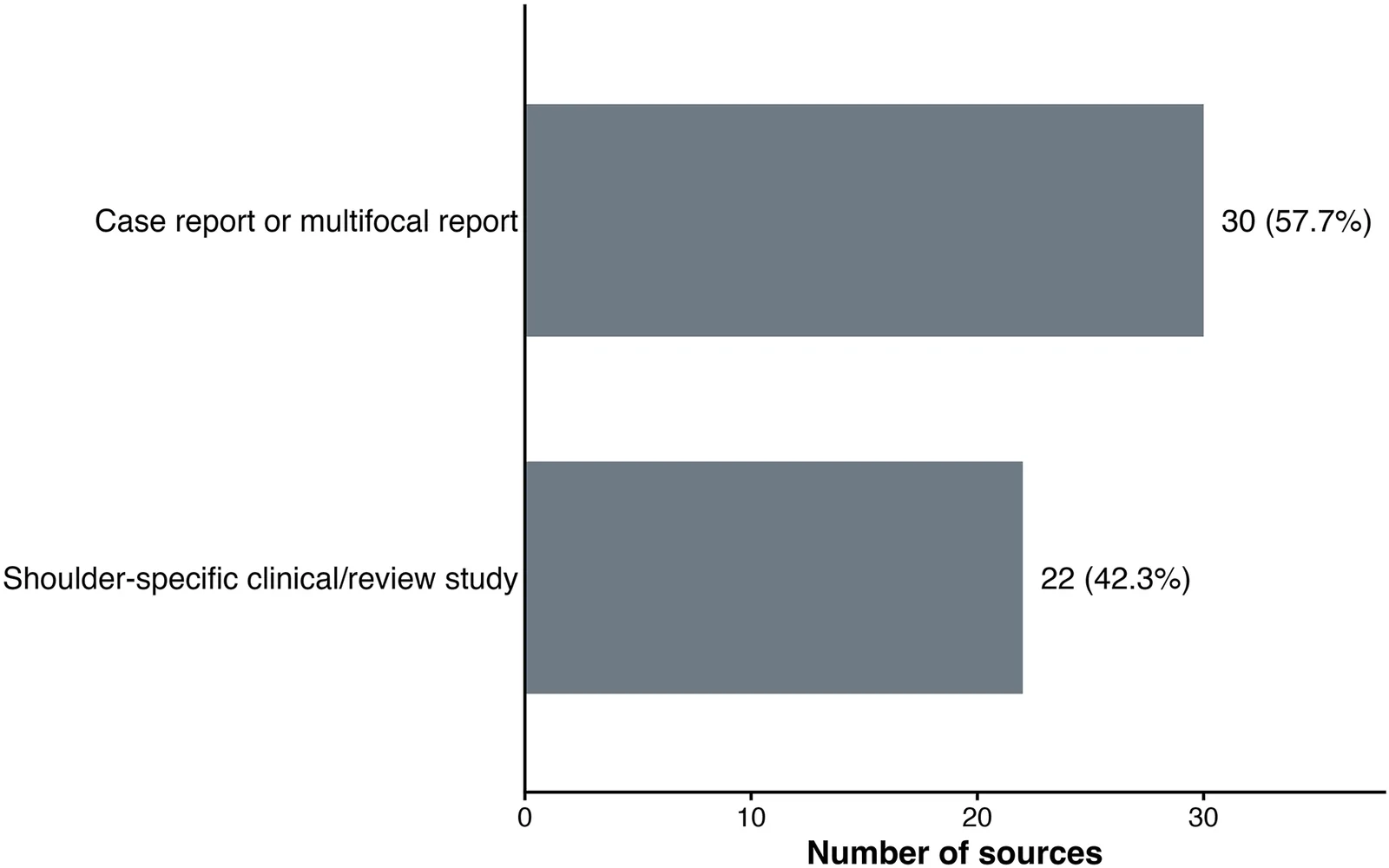
Distribution of included sources by evidence category. Values are shown as number of sources and percentage of the total included evidence set (n = 52).

Detailed characteristics of included studies and extracted variables are provided in Supplementary Table S1.^1^, ^21^, ^22^, ^23^, ^24^, ^25^, ^26^, ^27^, ^28^, ^29^, ^30^, ^31^, ^32^, ^33^, ^34^, ^35^, ^36^, ^37^, ^38^, ^39^, ^40^, ^41^, ^42^, ^43^, ^44^, ^45^, ^46^, ^47^, ^48^, ^49^, ^50^, ^51^, ^52^, ^53^, ^54^, ^55^, ^56^, ^57^, ^58^

### Corticosteroid dose reporting

Across the 52 included sources, corticosteroid dose reporting was inconsistent (Fig. 3). Quantified corticosteroid exposure was reported in 12 sources, partial or incomplete dose information was reported in 3 sources, and 9 sources provided no extractable corticosteroid dose data. Steroid exposure was mentioned but not quantified in 28 sources. Among shoulder-specific studies, Hernigou et al’s^7^ 2010 cohort provided the clearest quantitative shoulder-focused exposure data, recording corticosteroid dosage as cumulative, peak, and mean daily methylprednisolone-equivalent exposure; patients with humeral head osteonecrosis had a mean cumulative methylprednisolone-equivalent dose of 7,865 mg and a mean peak dose of 342 mg, but no reproducible dose threshold for disease development was proposed. In contrast, many shoulder-specific natural history and surgical outcome studies identified corticosteroid exposure as an etiologic factor but did not report cumulative dose, peak dose, daily dose, route, duration, or timing in a standardized manner.^4^^,^^6^^,^^10^^,^^12^^,^^14^^,^^18^ Case reports more often provided individual-level steroid exposure details, including intra-articular injection dose, oral methylprednisolone dose, cumulative prednisolone-equivalent exposure, and timing to symptom onset or diagnosis.^1^, ^2^, ^3^^,^^19^

**Figure 3:**
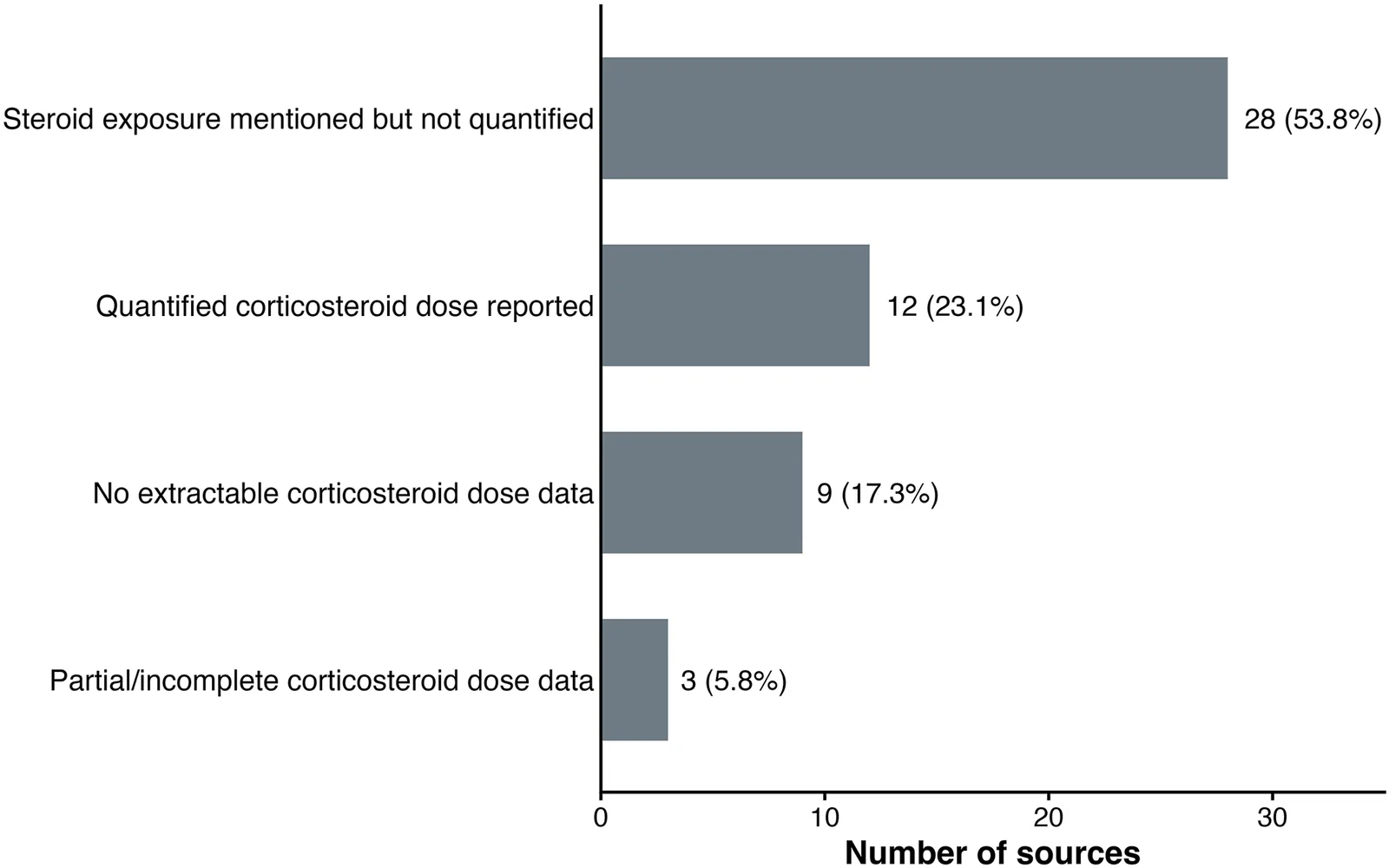
Corticosteroid dose reporting completeness across included sources. Values are shown as number of sources and percentage of the total included evidence set (n = 52).

### Evidence for dose-response or threshold

Evidence for a corticosteroid dose-response relationship in atraumatic humeral head osteonecrosis was limited. Within the shoulder-specific literature, quantitative evidence remained limited to observational comparison rather than threshold derivation. Although Hernigou et al’s^7^ 2010 cohort suggested greater corticosteroid exposure among patients who developed shoulder osteonecrosis, the study did not identify a clinically actionable cutoff that could be applied prospectively. Additional case reports and multifocal osteonecrosis reports described corticosteroid exposure preceding humeral head or shoulder involvement, but these reports were not designed to estimate dose-response relationships or threshold effects.^1^^,^^2^^,^^3^^,^^19^ Broader literature involving hip, transplant, severe acute respiratory syndrome (SARS), demyelinating disease, malignancy, and general osteonecrosis populations has reported associations between corticosteroid dose and osteonecrosis risk; however, findings from these broader populations cannot define a shoulder-specific threshold.^5^^,^^9^^,^^13^^,^^15^, ^16^, ^17^

### Summary of mapped evidence

Overall, the mapped evidence shows a consistent association between corticosteroid therapy and atraumatic osteonecrosis of the humeral head but insufficient exposure reporting to support a shoulder-specific threshold.^7^^,^^11^^,^^15^^,^^18^ Shoulder-focused studies generally treated steroid use as a categorical risk factor, whereas case reports and multifocal osteonecrosis reports more often described individual treatment histories without providing denominator-based risk estimates.^1^^,^^2^^,^^3^^,^^19^ Contextual non-shoulder studies support biological plausibility for a dose-related effect, but they cannot replace direct humeral head dose-response evidence.^5^^,^^9^^,^^13^^,^^16^^,^^17^ Together, Figures 2 and 3 demonstrate that the evidence base is broad enough to identify steroid therapy as an important risk factor, but too inconsistently reported to define a reproducible humeral head-specific dose threshold.

## Discussion

### Principal findings

This scoping review identifies a persistent reporting gap in the literature on steroid-associated osteonecrosis of the humeral head. Across the final 52-source evidence set, corticosteroids were repeatedly described as an important atraumatic risk factor, but exposure details were often incomplete. Most shoulder-specific reports documented steroid use as present or absent rather than reporting cumulative dose, peak dose, daily dose, treatment duration, route of administration, or timing from therapy to diagnosis.^11^^,^^12^^,^^15^^,^^18^ Consequently, the available evidence does not support a reproducible exposure threshold for atraumatic humeral head osteonecrosis.

### Interpretation of the evidence gap

The lack of a reliable threshold appears to reflect how the shoulder literature was designed rather than proof that dose is unimportant. Most humeral head studies focused on natural history, radiographic progression, staging, or surgical outcomes, rather than medication exposure assessment.^4^^,^^6^^,^^10^^,^^14^ As a result, steroid therapy was usually recorded as an etiologic category rather than a measurable exposure. This distinction matters because identifying steroids as a risk factor does not determine whether risk varies by cumulative dose, peak dose, daily dose, duration, route, pulse therapy, or timing of treatment.^5^^,^^15^ Without consistent exposure metrics, cross-study comparison remains limited and threshold inference is not possible.

### Relationship to broader corticosteroid-induced osteonecrosis literature

The broader corticosteroid-induced osteonecrosis literature suggests that dose may influence osteonecrosis risk, but the relationship varies by population, disease context, steroid regimen, and anatomical site. Contextual studies in renal transplantation, SARS-treated cohorts, malignancy, demyelinating disease, and mixed osteonecrosis populations have reported associations between osteonecrosis and cumulative dose, early high-dose exposure, or mean daily corticosteroid dose.^5^^,^^9^^,^^13^^,^^15^, ^16^, ^17^ These studies support biological plausibility for a dose-related effect. However, most of this evidence focuses on femoral head or multifocal osteonecrosis rather than isolated humeral head disease. Differences in vascular anatomy, mechanical loading, clinical presentation, imaging practices, and underlying disease populations limit direct extrapolation from hip-focused evidence to humeral head osteonecrosis.^8^^,^^11^ Therefore, broader steroid-dose literature can inform the rationale for concern but cannot define a shoulder-specific corticosteroid dose threshold.

### Clinical implications

Clinically, steroid therapy should remain an important consideration when evaluating atraumatic shoulder pain, particularly in patients with systemic inflammatory disease, transplant history, malignancy, demyelinating disease, recent severe respiratory illness treated with corticosteroids, prior high-dose therapy, or multifocal joint symptoms. However, current evidence does not support a numeric dose cutoff that can guide shoulder surveillance or early imaging. The absence of a defined threshold should therefore not be interpreted as evidence of low risk. Instead, clinicians should consider steroid history alongside symptom timing, coexisting osteonecrosis risk factors, multifocal pain, and the possibility of early disease that may be radiographically occult.

### Research implications

Future studies should report corticosteroid exposure in a standardized and reproducible manner. At minimum, studies of atraumatic humeral head osteonecrosis should report steroid type, route of administration, cumulative dose, peak dose, daily dose, treatment duration, use of pulse therapy, timing from corticosteroid initiation to symptom onset, and timing from corticosteroid initiation to imaging diagnosis. Studies should also distinguish isolated humeral head osteonecrosis from multifocal disease because many shoulder cases occur alongside femoral head or other skeletal involvement.^2^^,^^3^^,^^11^^,^^19^ Prospective or multicenter cohort studies would be needed to determine whether corticosteroid dose independently predicts humeral head osteonecrosis after accounting for underlying disease, transplant status, systemic inflammatory conditions, alcohol use, sickle cell disease, malignancy, and other confounders.

### Limitations

This review has several limitations. First, although the search included PubMed/MEDLINE, Embase, the Cochrane Library, Google Scholar, and manual reference-list screening, Google Scholar was used as a supplementary source; the updated search retrieved 35 records, of which 29 unique records were screened after duplicate and overlap removal. Second, the evidence base was heterogeneous across study design, patient population, steroid indication, imaging approach, disease stage, anatomical site, and outcome reporting, limiting direct comparison between studies. Third, many shoulder-specific reports were retrospective case series or surgical outcome cohorts, which are vulnerable to selection bias and often over-represent patients with advanced disease.^4^^,^^12^^,^^14^^,^^18^ Fourth, steroid treatment was frequently reported incompletely or categorically, preventing formal dose-response analysis or meta-analysis.^7^^,^^15^

## Conclusion

Current shoulder-specific literature supports corticosteroid exposure as an important risk factor for atraumatic osteonecrosis of the humeral head, but it does not provide sufficient standardized dose reporting to define a reliable corticosteroid dose threshold. Most shoulder-focused studies report corticosteroid exposure as an etiologic category rather than as a quantifiable exposure, limiting assessment of cumulative dose, peak dose, daily dose, route, duration, or timing. Although broader hip, transplant, malignancy, SARS-related, demyelinating disease, and multifocal osteonecrosis literature suggests that corticosteroid dose may influence osteonecrosis risk, these findings cannot be directly applied to the humeral head. Future studies should use standardized corticosteroid exposure reporting and distinguish isolated humeral head disease from multifocal osteonecrosis to enable meaningful dose-response analysis.

## Disclaimers:

Funding: No funding was disclosed by the authors.

Conflicts of interest: The authors, their immediate families, and any research foundations with which they are affiliated have not received any financial payments or other benefits from any commercial entity related to the subject of this article.

## Declaration of generative AI and AI-assisted technologies in the writing process

During the preparation of this work, the authors used ChatGPT to assist with coding for figure generation in RStudio. After using this tool, the authors reviewed and edited the content as needed and took full responsibility for the content of the manuscript.

## Supplementary Data

Supplementary Table S1

PRISMA ScR Checklis
