# Supplementary material for: Corticosteroid dose reporting in atraumatic osteonecrosis of the humeral head: a scoping review
Source: JSES Rev Rep Tech. 2026 Sep 4;7(1):100877. doi: 10.1016/j.xrrt.2026.100877 (PMC13654033; doi:10.1016/j.xrrt.2026.100877)
Supplement: Supplementary file 1 [file mmc2.docx]

PRISMA-ScR Checklist

Manuscript title:

Corticosteroid Dose Reporting in Atraumatic Osteonecrosis of the Humeral Head: A Scoping Review

Authors:

Thomas Ciavarro, Patrick Carroll, Amr Elmaraghy

| **PRISMA-ScR item** | **Checklist topic** | **Where addressed in manuscript** |
| --- | --- | --- |
| Title | Identify the report as a scoping review | Title page |
| Abstract | Provide summary of background, purpose, methods, results, and conclusion | Abstract |
| Rationale | Describe why the review was conducted | 1. Introduction |
| Objectives | State the review question/objectives | End of 1. Introduction; 2.2 Research question |
| Protocol and registration | State whether protocol exists/was registered | 2.1 Study design |
| Eligibility criteria | Define included/excluded sources | 2.3 Eligibility criteria |
| Information sources | Describe databases and other sources searched | 2.4 Information sources and search strategy |
| Search strategy | Present full search strings | 2.4 Information sources and search strategy |
| Selection of sources of evidence | Describe screening process | 2.5 Study selection |
| Data charting process | Describe extraction/charting process | 2.6 Data charting |
| Data items | List extracted variables | 2.6 Data charting; Supplementary Table 1 |
| Critical appraisal | State whether appraisal was performed | 2.7 Synthesis of results / add “No formal critical appraisal was performed, consistent with scoping review methodology.” |
| Synthesis of results | Describe how evidence was grouped/synthesized | 2.7 Synthesis of results; 2.8 Dose-reporting classification |
| Selection of sources | Report screening/inclusion results | 3.1 Study selection; Figure 1 |
| Characteristics of sources | Describe included evidence | 3.2 Characteristics of included evidence; Supplementary Table 1 |
| Results of individual sources | Present relevant extracted data | Supplementary Table 1 |
| Synthesis of results | Summarize mapped findings | 3.3–3.5 Results |
| Summary of evidence | Summarize main findings | 4.1 Principal findings; 5. Conclusion |
| Limitations | Discuss limitations of review process/evidence base | 4.6 Limitations |
| Conclusions | Provide interpretation and implications | 5. Conclusion |
| Funding | Report funding sources | Funding statement |
